# Metabolic Rewiring in Glioblastoma Cancer: *EGFR*, *IDH* and Beyond

**DOI:** 10.3389/fonc.2022.901951

**Published:** 2022-07-14

**Authors:** Abdellatif El Khayari, Najat Bouchmaa, Bouchra Taib, Zhiyun Wei, Ailiang Zeng, Rachid El Fatimy

**Affiliations:** ^1^ Institute of Biological Sciences (ISSB-P), Mohammed VI Polytechnic University (UM6P), Ben-Guerir, Morocco; ^2^ Institute of Sport Professions (IMS), Ibn Tofail University, Avenida de l’Université, Kenitra, Morocco; ^3^ Research Unit on Metabolism, Physiology and Nutrition, Department of Biology, Faculty of Science, Ibn Tofail University, Kenitra, Morocco; ^4^ Shanghai Key Laboratory of Maternal Fetal Medicine, Shanghai First Maternity and Infant Hospital, School of Medicine, Tongji University, Shanghai, China; ^5^ Department of Cancer Biology, University of Texas MD Anderson Cancer Center, Houston, TX, United States

**Keywords:** GBM, genetic alteration, metabolic genes, glycolysis, glioma therapy

## Abstract

Glioblastoma multiforme (GBM), a highly invasive and incurable tumor, is the humans’ foremost, commonest, and deadliest brain cancer. As in other cancers, distinct combinations of genetic alterations (GA) in GBM induce a diversity of metabolic phenotypes resulting in enhanced malignancy and altered sensitivity to current therapies. Furthermore, GA as a hallmark of cancer, dysregulated cell metabolism in GBM has been recently linked to the acquired GA. Indeed, Numerous point mutations and copy number variations have been shown to drive glioma cells’ metabolic state, affecting tumor growth and patient outcomes. Among the most common, IDH mutations, EGFR amplification, mutation, PTEN loss, and MGMT promoter mutation have emerged as key patterns associated with upregulated glycolysis and OXPHOS glutamine addiction and altered lipid metabolism in GBM. Therefore, current Advances in cancer genetic and metabolic profiling have yielded mechanistic insights into the metabolism rewiring of GBM and provided potential avenues for improved therapeutic modalities. Accordingly, actionable metabolic dependencies are currently used to design new treatments for patients with glioblastoma. Herein, we capture the current knowledge of genetic alterations in GBM, provide a detailed understanding of the alterations in metabolic pathways, and discuss their relevance in GBM therapy.

## Introduction

Over the past few years, significant advances in understanding glioma at the molecular level have greatly improved our understanding of the genetic alterations that characterize this heterogeneous brain tumor. Since 2016, with the revised World Health Organization (WHO) classification of tumors of the Central Nervous System (CNS), different glioma entities are defined not only by histological features but also by genetic and molecular markers (1). Besides, the classification based on genetic alterations has helped distinguish primary and secondary gliomas, unlike that based on histopathological features (1, 2). Moreover, recent studies have shown that the genetic characteristics adopted in conjunction with histopathological data have remarkably improved the diagnosis and prognosis of gliomas (3), with the benefit of providing new insights into targeted strategies in the treatment of malignant gliomas. Nevertheless, the fourth-grade glioma, also known as glioblastoma multiform (GBM), has disposed of a particular challenge within the clinical setting due to its unique and distinctive cellular heterogeneity. The frequent occurrence of genetic and epigenetic alterations has made GBM immune to all conventional cancer therapies ranging from resection to chemotherapy and radiation therapy (Cancer Genome Atlas Research Network, 2008) (4). Alongside, GBM tumors rapidly develop therapeutic resistance, making this cancer intractable to date. Most of the available pre-clinical and clinical treatment strategy stages target host immune response stimulation to decrease tumor proliferation and invasion through immune and/or gene therapies (3, 4).

To date, therapies that target the angiogenic nature of the tumor, such as the use of monoclonal antibodies for Vascular Endothelial Growth Factor (VEGF) (Bevacizumab), have displayed some promising results in the clinical trials (5). Besides using alkaline agents such as Temozolomide and other targeted therapies like the inhibitors of growth factors, transcriptional and translational pathways coupled with radiation therapy are finding their way into pre-clinical and clinical trials (6). Further, the Epidermal Growth Factor Receptor (EGFR), a critical factor in tumor malignancy in several cancer types, including glioblastoma, has emerged as one of the most explored drug targets in cancer therapeutics over the past 20 years (7). For example, several EGFR inhibitor-based approaches are currently used to treat patients with EGFR-positive tumors, notably non-small cell lung cancer. In GBMs, abnormal *EGFR* activation due to somatic mutations and *EGFR* gene amplification is a reliable feature incriminated in various pathological processes. Recently, metabolic rewiring of GBM cells was linked mainly to these alterations, and a considerable body of evidence considered them among the most potentially targetable alterations in GBM (8, 9). However, the outcome of all available therapeutic options against GBM remains non-universal due to the disease’s rapid variations at the molecular, genetic, and epigenetic levels, which are unique across individuals (10).

Therefore, therapeutic solutions are driven primarily toward developing a personalized treatment. This emerges the need to acquire an in-depth understanding of gliomas’ molecular programming and reprogramming, including several deletions, amplifications, and mutations followed by metabolic alterations (10).

Metabolism-related gene signatures, which may more effectively predict patient prognosis, are still lacking in gliomas, especially in GBM cancer. Understanding the malignant phenotype of gliomas requires knowledge of these metabolic alterations to decipher the mechanisms underlying the complex relationship between molecular aberrations, metabolism profile, and tumor behavior. Indeed, recent evidence increasingly highlights the high frequency of alterations in metabolic genes, also revealing a strong correlation between the grade of malignancy and the occurrence of these alterations (11, 12). The extensive use of glucose by cancer cells for energy generation through aerobic glycolysis, a phenomenon is known as the “Warburg effect,” as opposed to mitochondrial oxidative phosphorylation (OXPHOS), is responsible for the rapid generation of adenosine triphosphate (ATP) and adaption to the hypoxic tumor environment (13). In the case of GBM, it is now clear that malignant cells adapt their bioenergetic metabolism to overcome the fluctuations/constraints in tumor microenvironmental conditions and maintain their high proliferative characteristic. Thus, the rate of aerobic glycolysis and OXPHOS is closely associated with tumor stage, microenvironment, and specifically activated oncogenes (14). The balance between high glycolysis and OXPHOS drives the bioenergetics of the tumor, enhancing the malignant processes such as cell proliferation and invasion.

Moreover, in GBM, several genes associated with glycolysis have been suggested to correlate with tumor proliferation, invasion, angiogenesis, and chemoresistance. Likewise, fatty acids and other lipid species are thought to contribute to the malignancy of GBM (15). In addition to their bioenergetic role, several proofs have shown the effective implication of glycolysis and lipid metabolism in modulating the tumor microenvironment and facilitating adaptation to hypoxic conditions. Also, inhibitors of some metabolic enzymes involved in these pathways have shown promising outcomes in enhancing radio-sensitivity in patients with gliomas (16, 17). However, integrated analyses have evidenced that glycolysis and lipid alterations are significantly associated with worse overall survival of patients (11, 18), suggesting the involvement of these pathways in the aggressiveness of glioblastoma. Therefore, shedding light on these altered metabolic processes is urgently needed to harness the metabolic clues provided by the available knowledge and the ongoing efforts to develop new therapeutic approaches for glioma cancers.

The purpose of this review is to capture an overview of the current understanding of the hallmark genetic alterations in GBM, and explore compelling evidence for the common dysregulated metabolic pathways such as lipid synthesis and glycolysis, with a particular focus on the complex interplay between genomic aberrations and altered cellular metabolism in GBM. Furthermore, the significance of established metabolic dependencies as emerging targets in advancing therapeutic solutions against GBM is thoroughly discussed.

## Major genetic alterations in GBM

GBM has been known as a highly nonhomogeneous cancer (3, 4), and it is obscure how genomic alterations conduce to this phenotype. With the growing effectiveness of genetically targeted precision therapy for other tumor malignancies (19), several prior studies have been first interested in investigating and identifying numerous credibly targetable mutations as well as copy number changes and epigenetic variants that are usually common in GBMs. In this respect, the new classification system, merging classical histologic classification and molecular genetic data alterations, divided GBMs into primary GBMs (*IDH (Isocitrate dehydrogenase)* wild-type) and secondary GBMs (*IDH* mutant) (10–12), allowing as well a better characterization of gliomas (20, 21). Furthermore, specific genetic rearrangements are believed to have clinical relevance and are further related to GBMs class II (20). Besides, in recent years, molecular alterations in metabolic gene *IDH 1/2* have received much attention. Admittedly, mutations of *IDH1/2* are considered early events in tumorigenesis (22) and may reveal tumor vulnerabilities that can be exploited for potential therapeutics (23). Significantly, in adults, molecular alterations in this class of metabolic gene are now recognized as a defining molecular event commonly frequent in the secondary GBMs (50%), which are also known by *O6-methylguanine-DNA methyltransferase promoter methylation (MGMT)* (75%) (20), as well as in the majority of lower-grade astrocytoma (WHO grade II and III gliomas) (80%), which are also both reported by tumor protein (*TP53)* mutations and functional loss (mutation or deletion) *of α-Thalassemia/Mental Retardation Syndrome X-linked (ATRX) (* 24, 25). In contrast, primary GBM, which are nearly all *IDH Wild-type*, are typically displaying *epidermal growth factor receptor* (*EGFR)* and *mouse double minute 2 homolog (MDM2)* amplification or mutation, *cyclin-dependent kinase inhibitor 2 A/B (CDKN2A/B)* deletion, *phosphatase, and tensin homolog* (*PTEN)* loss *and neurofibromatosis type 1(NF1)*, and *telomerase reverse transcriptase (TERT)* promoter mutations, along with the gain of chromosome 7 and loss of heterozygosity at q10 (LOH 10q) (chromosome 10) (25). Interestingly, such genetic alterations permit an accurate distinction between primary and secondary GBMs, even though they are histologically indistinguishable. Additionally, primary GBMs, representing about 90% of all GBM subsets, show other less-common mutations and/or deletions in genes such as *TP53, PDGFRA, EGFRvIII*, *PIK3CA, PIK3R1, RB1, H3F3A, MET, CDK4, CDK6*, and *MDM4* (24–26).

Likewise, a recent investigation through mutually exclusive gene set analysis (MEGSA) reported the alterations in Spectrin Alpha Erythrocytic 1 (*SPTA1)* and *Capping Actin Protein of Muscle Z-Line Subunit Alpha 2 (CAPZA2)* are linked to GBMs. This investigation suggests that mutated *SPTA1* may be related to abnormal cell proliferation and apoptosis, while amplified *CAPZA2* negatively regulates cell invasion (27). Moreover, *H3K27M* (A methionine substitution of lysine at residue 27 of histone H3) mutation, regular in pediatric diffuse midline glioma, has also been recognized in malignant adult glioblastoma, with mutual exclusion to *IDH1/2* alterations (28).

**Figure 1:**
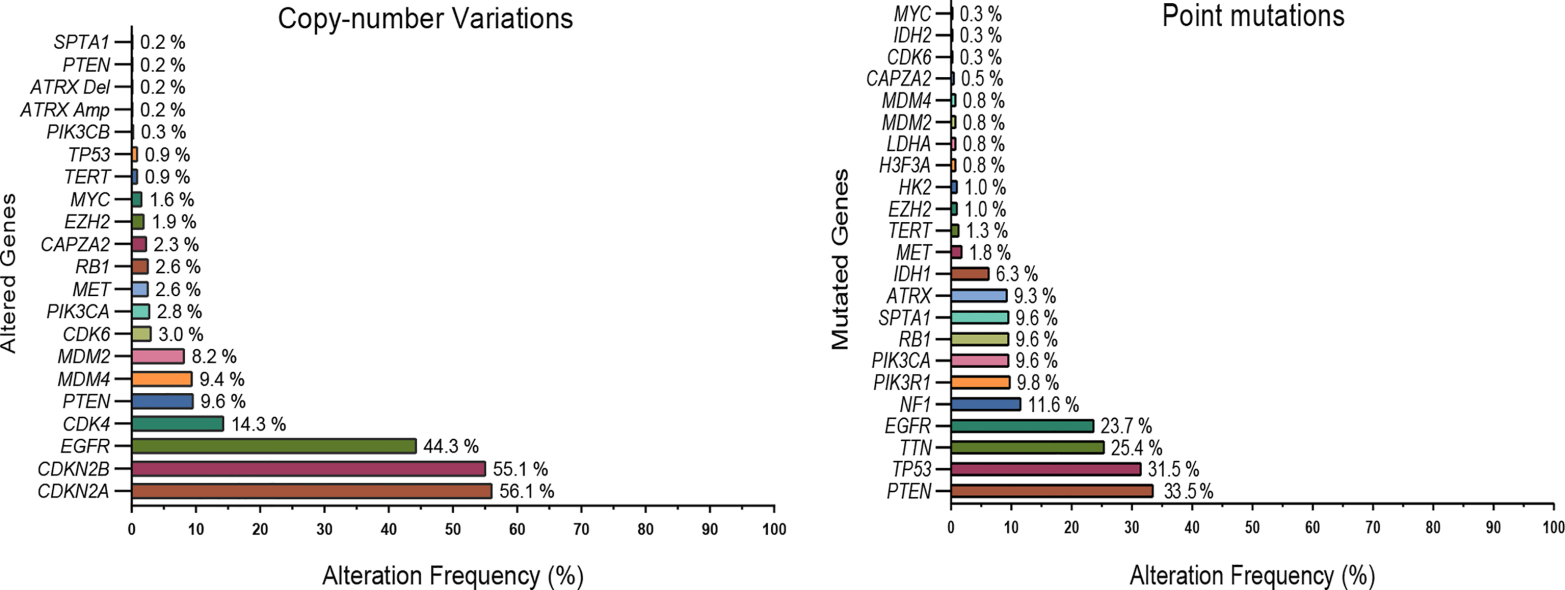
Commonly mutated genes and copy number variations in GBMs. Data (592 patients) from the TCGA dataset (Glioblastoma multiforme; TCGA- PanCancer Atlas) were explored using the cBioPortal platform. For clarity, only a subset of highly altered and metabolic genes are shown. *EGFR, PTEN, TERT*, and several metabolic genes such as *IDH 1/2*, *HK2, MYC, LDHA, EZH2* were frequently altered in glioblastoma tumors.

Considerably, understanding the metabolic reprogramming (MR) of aggressive gliomas, a phenomenon that could potentially perform as a hallmark of cancer progression, has potential functions for therapy in tandem with biomarkers. To demonstrate how tumor heterogeneousness and therapeutic resistance promote this GBM phenotype, we investigated numerous recent studies exploring the genomic and epigenetic alterations in these tumors. This examination has also revealed that common pathways are altered in GBM, specifically metabolism pathways (**Figure 1**).

Commonly, it is known that mutations that co-occur in driver genes trigger different cell signaling pathways that work together in carcinogenesis. Also, driver genes that display a mutually exclusive mutation pattern can have a redundant oncogenic function (29). For example, similar co-occurrence patterns that have been shown to collaborate in model organisms consist of alterations in KRAS and TP53 in pancreatic cancer, ERG translocations and PTEN deletions in prostate cancer, APC and KRAS in CRC, or MYC and TP53 in various cancer types (4, 30–34). Therefore, biological insights into the concurrent interactions of driver genes could also have crucial implications for improving novel therapeutic approaches. The common co-targeting of co-occurring and collaborating driver mutations or their related signaling pathways has long been a goal in preclinical and clinical research.

## Genetic link to metabolism in gliomas

MR of tumors, an energetic process for maintaining the reduction-oxidation balance and macromolecular biosynthesis, is considered a hallmark of cancer progression required for cell growth, proliferation, and migration (18, 35). Therefore, malignancy cells must improve metabolic pathways by utilizing available nutrients to maintain energy demand, redox balance, and biosynthesis to prolong and achieve their proliferative potentiality. However, the altered mechanisms behind this metabolism shift remain complex and not completely understood. Nevertheless, a growing number of studies are underway to explore the potential of a metabolism-targeting strategy as a novel and promising approach against gliomas (36). However, accumulating evidence demonstrates that genetic alterations partially drive gliomas’ cell metabolism and contribute to the aggressive nature of some cancers like GBM.

### IDH Mutations Shift Gliomas Metabolism Through D-2-Hydroxyglutarate

The most studied example linking genetic alterations to gliomas metabolic changes is the acquired mutation in *IDH1* and/or *IDH2* genes. While *IDH* mutation has been detected in several cancer types and reported to have the same primary effects in those malignancies. However, gliomas remain the only cancer in which the *IDH1/2* mutation constitutes a specific feature representing a propitious prognostic marker (37, 38). The natural enzymatic reaction catalyzed by the wild-type *IDH1* and *IDH2* is an oxidative decarboxylation of isocitrate into α-ketoglutarate (α-KG), and the production of the reduced form of the nicotinamide adenine dinucleotide phosphate (NADPH) (39). However, *IDH* mutation leads to the consumption of α-KG and NADPH to produce an oncometabolite, D-2-hydroxyglutarate (D2HG), directly involved in G-CIMP phenotype inducing and aberrant DNA methylation in gliomas (40, 41). In addition, D-2-hydroxyglutarate was reported as a competitive inhibitor for some α-ketoglutarate-dependent dioxygenases (42). Therefore, the pro-methylation effects of the *IDH* mutation may be linked to the extent to which a specific cell expresses the affected dioxygenases. For instance, in the study conducted by Unruh et al. (2019) aiming to determine the methylation and transcription patterns in *IDH* mutant gliomas, authors showed that of 365,092 analyzed CpG sites, about 19% were hypermethylated in gliomas with *IDH* mutation compared to wild-type gliomas (38). However, in acute myeloid leukemia, cholangiocarcinoma, and melanoma, only 3%, 4%, and 2% of CpG sites were hypermethylated in the *IDH* mutant cancers, respectively. Moreover, the hypermethylation degree was lower in mature astrocytes than in the undifferentiated neural progenitor cells, which may justify the importance of the *IDH* mutation as a helpful prognostic marker in gliomas (38).

D-2-hydroxyglutarate was also reported to inhibit the branched-chain amino acid aminotransferase (BCAT) transaminases and thus rendered gliomas cells addicted to glutamine and more sensitive to glutaminase inhibition (36, 43). Of note, primary GBM and other wild-type IDH gliomas display a similar addiction to glutamine, suggesting different players for the glutamine-dependent energy pathway observed in gliomas (36). Moreover, the metabolic dysregulations associated with *IDH* mutations could also be extended to glycolysis, TCA cycle metabolism, and phospholipid and Reactive oxygen species (ROS) production (44–46).

### *EGFR* Mutation Promotes Glycolysis, Glutaminolysis, and Lipogenesis *via* mTOR and MYC in Glioblastoma

A growing body of proof suggests that oncogenic mutated epidermal growth factor receptor (*EGFR*) favors an MR to glycolysis, which is conceivably reversed *via* EGFR tyrosine-kinase inhibitors (TKIs), thereby inhibiting cancer cell growth.

Indeed, tyrosine phosphorylation is a metabolic process of multicellular organisms crucial for signal transduction. Tyrosine phosphorylation is, therefore, essential for many cellular mechanisms, such as differentiation, proliferation, migration, and survival (47).

Receptor tyrosine kinases (RTKs) are membrane-spanning proteins activated by the binding of a specific ligand to their extracellular domain. The change following this reaction led to the intracellular catalytic domain initiation, enabling the proteins occupation to trigger a signaling pathway in response to a specific cellular demand (48, 49). The epidermal growth factor receptor (*EGFR*) was highly altered in gliomas and the most amplified in GBM (50). As a result, the *EGFR* amplification and mutation led to several cellular processes modification, contributing to tumorigenesis and progression.

Despite the implication of proto-oncogene family *MYC* in several metabolic processes, including nucleotide synthesis, lipogenesis, glucose transport, and glycolysis, its role in some gliomas, such as adult GBM, for which the *MYC* is rarely amplified, was not fully understood until recently (8, 49). MYC regulates the expression of several genes involved in glucose transport and metabolism. This includes the glucose *transporter GLUT1, hexokinase 2* (*HK2*), the *muscle isoform of phosphofructokinase* (*PFKM*), *enolase 1* (*ENO1*), and lactate dehydrogenase A (*LDHA*) (51, 52), which leads to the Warburg effect characterized by an increase in the glucose uptake and its fermentation to lactate (53). Several works have revealed that *EGFR* mutation and amplification modulate GBM metabolism through *MYC via* three complementary mechanisms. This includes **(1)** EGFRvIII impacts an isoform arrangement by modulating the heterogeneous nuclear ribonucleoprotein A1 (hnRNPA1). This protein leads to the splicing of the Delta max, which is an MYC-interacting partner that promotes glycolytic metabolism (54). **(2)** Simultaneously, EGFRvIII increases glycolysis in GBM via the mTORC2 signaling that regulates the cellular level of c-Myc through acetylation of forkhead box protein O1 (FoxO1) and FoxO3, leading to an upregulated expression of MYC protein (55). **(3)** The hyper-activated EGFRvIII promotes the transcription of *SOX9* and *FOXG1*, which is implicated in regulating the MYC-dependent transcription (56). All the proofs, as mentioned above, show the pivotal player of MYC for the EGFRvIII-dependent tumorigenesis.

EGFR was also reported to influence glutamine metabolism in GBM cells. Indeed, after glutamine absorption by cancer cells, glutaminase converts it to glutamate, which enters the mitochondria or the cytoplasm to maintain the operational TCA cycle. Indeed, glutamate is an indispensable molecule in tumor cells due to its use as a forerunner to produce α-KG, which enters the TCA cycle to generate carbon and nitrogen for the cancer cells (57–59). The conversion of glutamate to the α-KG is catalyzed by glutamate dehydrogenase (GDH1). Furthermore, the study conducted by Yang et al. (2020) showed that phosphorylated ELK1 activated by the EGFR signaling pathway induced the transcription of GDH1, leading to the increase of glutamine metabolism (59).

Collectively, these studies show that EGFR is a critical factor in gliomas’ development and progression through its implication in the regulation of the proto-oncogene *MYC* family and the activation of the glutamine metabolism through the activation of ELK1.

Additionally, glioma cells involve further mutations to reprogram their metabolism and support high-energy demand. For example, *PTEN* loss has been seen to upregulate hexokinase 2 (HK2) and activate the autophosphorylated PGK1, promoting aerobic glycolysis in GBM (36, 60). Also, *TERT* promoter mutation is thought to regulate lipid metabolism through a histone H3K27 methyltransferase (EZH2), suggesting an essential role for *TERT* in MR of GBM cells (61).

## Epigenetic landscape and non-coding RNAs in GBMs

In addition to genetic alterations, accumulated epigenetic events play a fundamental role in GBM pathobiology simply as associated with poor prognosis. Therefore, each new epigenetic event might be a valuable marker for glioblastoma prognosis. Furthermore, GBMs display intra- and inter-tumor heterogeneity, a phenomenon in which diverse cells inside the same tumor procure distinct epigenetic and genomic mutations (36, 62).

Numerous studies showed the implication of this phenomenon, one of the major factors of therapeutic resistance to anticancer therapies, to drive tumor genomics landscape misapprehension, which is a crucial fundamental confrontation towards personalized medicine and biomarker advancement.

Accordingly, large-scale genomic studies have described many epigenetic defects involved in GBM, including changes in DNA methylation profile, histone post-transcriptional modifications, and chromatin remodeling (63). However, chronicles of mutations in several epigenetic regulator genes have been reported in GBM. Notably, mutations in *IDH 1/2*, *EGFR, H3F3A, MLL2-4, HDAC2, HDAC9, KDM4D, KDM5A/B/C*, and *KDM6A/B* have been observed to promote DNA and histone methylation and modify chromatin state, thereby inducing aberrant modifications in the epigenetic pattern and consequently affecting the gene expression profile in GBM cells (10, 64). Moreover, earlier records have shown evidence for CpG island methylator phenotype (CIMP) in GBM. These studies further report the association of GBM with frequent hypermethylation at specific loci, especially at the promoters of several genes involved in GBM pathologies such as *MGMT*, *CDKN2A^-^* ^p14ARF^ and *CDKN2A* ^-p16INK4a^, *RB*, and *TIMP-3*, which have direct implications in DNA repair, cell cycle regulation, tumor suppression, and inhibition of apoptosis, respectively (63, 65–67).

Given the critical role of non-coding RNAs (ncRNAs) as genetic regulators-controlling gene expression at the epigenetic level, microRNAs (miRNAs), a class of short ncRNAs, have recently emerged as key players in glioma pathogenesis. Recent studies revealed that approximately 351 miRNAs are dysregulated in GBM (68), and some of them have been associated with acquired malignant phenotypes such as tumor growth, proliferation, invasion, and angiogenesis. Among others, miRNA-21, miRNA-10b, miRNA-7, miRNA-100, miRNA-296, miRNA-210-3p, miRNA-128, and miRNA-221 are the prominent candidates that have been studied and investigated for miRNA-based therapeutic strategies in GBM (68–72). Alongside miRNAs, long ncRNAs (lncRNAs) have also been observed to control many cellular processes in glioma cells predominantly associated with GBM and its malignancy (68, 73, 74). Moreover, amassing proof approved the incrimination of miRNAs and LncRNAs as mediators in energy metabolism reprogramming of GBM tumors.

**Figure 2:**
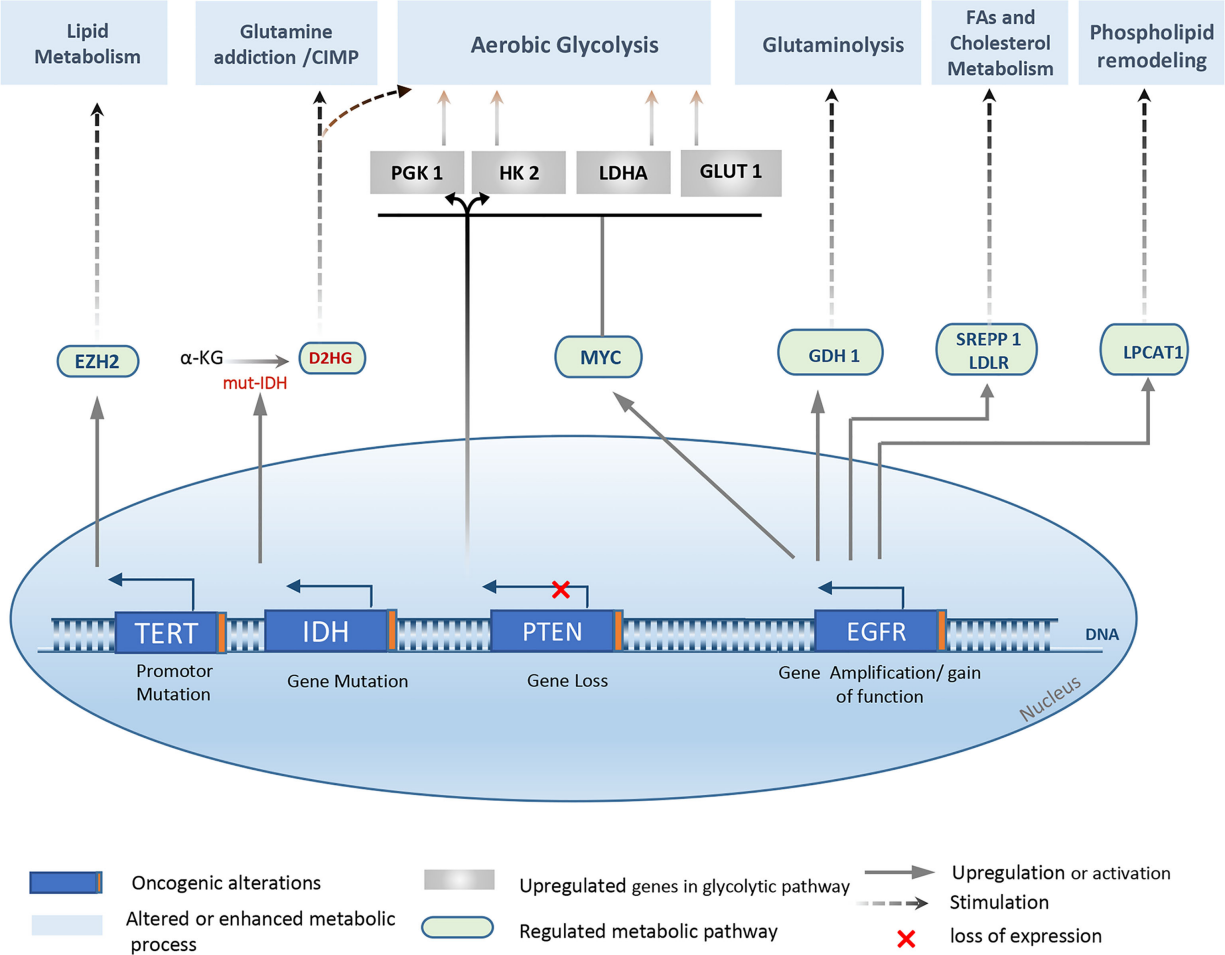
Overview of the major genetic alterations that drive metabolic reprogramming in Gliomas. Metabolic changes observed in gliomas and especially in glioblastoma are associated with several genetic abnormalities, mainly, *IDH ½* mutation*, EGFR* amplification, mutation, or EGFRvIII activation. Also, PTEN loss, and TERT promoter mutation. Various metabolic pathways are affected, yielding a new metabolic profile that supports the high proliferative characteristic, cell adaption, and tumor progression. EZH2, Enhancer of zeste homolog 2; D2HG, d-2-hydroxyglutarate; SREPP 1, sterol regulatory element-binding protein 1; LPCAT1, lysophosphatidylcholine acyltransferase 1; LDLR, glutamate dehydrogenase 1; LDHA, Lactate dehydrogenase A; GDH 1, glutamate dehydrogenase 1; PGK 1, phosphoglycerate kinase 1.

In addition to genetic and epigenetic variability among patients, three major molecular signaling pathways are altered in GBM: Growth Factor RTK pathway, Retinoblastoma (RB) pathway, and TP53 pathway (10, 63, 75). As a result, impaired phenotypes potentially contribute to different processes relevant to gliomagenesis, such as cell transformation, angiogenesis, invasion, and metabolic reprogramming (**Figure 2**).

Recently, a couple of Glutaminase isoenzymes have been proven to be linked to the expression of multiple miRNAs and could potentially promote tumor development or inhibition through various miRNA-mediated pathways (76). In GBM cells, miR-1297 repressed cell proliferation and glycolysis *via* targeting KPNA2 (77), a high oncogene in glioma tissues that was proven to be a high promoter of glycolytic metabolism in GBM cells as well (78). The downregulation of miR-1297 and upregulation of KPNA2 in glioma implied a regulatory association between them (78, 79).

Moreover, LncRNAs are considered an actor in cancer progression as miRNA decoys or targets. Furthermore, LncRNA HOX transcript antisense intergenic RNA (HOTAIR) was further observed upregulating the glutaminase expression level, which is crucial for glutamine metabolism and coming oncogenic approach. For instance, Liang Liu et al. (2018) reported the regulation function of HOTAIR on glutaminase (GLS) expression through sponging miR-126-5p in a competing endogenous RNA axis HOTAIR/miR-126-5p/GLS that is implicated in glioma progression (80). Similarly, It has been demonstrated that LINC00174 contributes to the carcinogenesis of glioma and further promotes glycolysis *via* miR-152-3p modulation, involving GLUT1 (SLC2A1) overexpression (81). Furthermore, He et al. also revealed that lncRNA UCA1/miR-182/PFKFB2 modulated glioblastoma-associated stromal cell-mediated glycolysis (82). In addition, referring to the existing literature, the oncogenic lncRNA PCAT19 and CARD8-AS1 were positively correlated with tryptophan metabolism and MDSC infiltration, suggesting their immunosuppressive function in the tumor microenvironment is potentially mediated by promoting immunosuppressive metabolism (83–85).

## Glycolysis in gliomas

From the previous discussion, the Warburg effect represents a hallmark of different cancer types, including GBM. Indeed, this phenomenon is responsible for shifting glucose metabolism toward aerobic glycolysis to produce ATP and other metabolites required for cell proliferation, invasion, and adaptation to the hypoxic surrounding microenvironment (86). In line with this, it has been reported that GBM cells exhibit an increased glucose uptake compared to normal astrocytes and utilize cytosolic fermentation rather than mitochondrial oxidation, thereby producing large amounts of lactate, which is subsequently released into the extracellular space (87). Also, GBM-derived extracellular vesicles (GBM EVs) appear to play a pivotal role in reprogramming transformed cells toward a glycolytic state through horizontal mRNA transfer (extracellular RNA) and upregulation of several metabolic pathways (88).

Even though numerous reports have suggested that this unbalanced metabolic balance linking glycolysis with oxidative phosphorylation in gliomas cells is due solely to mitochondrial disorder (89), more current proof from *in vitro* and *in vivo* studies has also shown that either acquired genetic mutations or hypoxia increase glycolytic ratio and as a result of this re-modulate cellular energy metabolism of GBM cells.

Furthermore, immune cells, particularly macrophages, contribute to this metabolic adaptation by secreting interleukin-6 (IL-6) in the tumor microenvironment, phosphorylating thereby PGK1 and enhancing glycolysis in GBM cells (90). Moreover, GBMs are metabolically heterogeneous tumors and often show high metabolic plasticity depending on micro-environmental conditions (91). Hence, high rates of glycolysis occur in the tumor's central region whereas the peripheral region predominantly utilizes oxidative phosphorylation. Likewise, glioblastoma stem cells (GSCs) are less glycolytic than differentiated cells (92). Altogether, the last pieces of evidence support the crucial role of micro-environmental conditions, alongside the genetic alterations, in modulating metabolic pathways and regulating tumor growth (93).

However, recent reports have revealed a remarkable correlation between glycolysis and gliomas malignancy, whereby glycolytic profile was closely associated with poor prognosis and overall survival in patients with GBM (62, 94). Additionally, *in vitro* studies have demonstrated that inhibiting glycolysis sensitizes glioblastoma cells and significantly enhances the efficacy of radiotherapy and chemotherapy (temozolomide) against these highly malignant tumors (16, 17).

### Advantages of Aerobic Glycolysis in GBM

**Figure 3:**
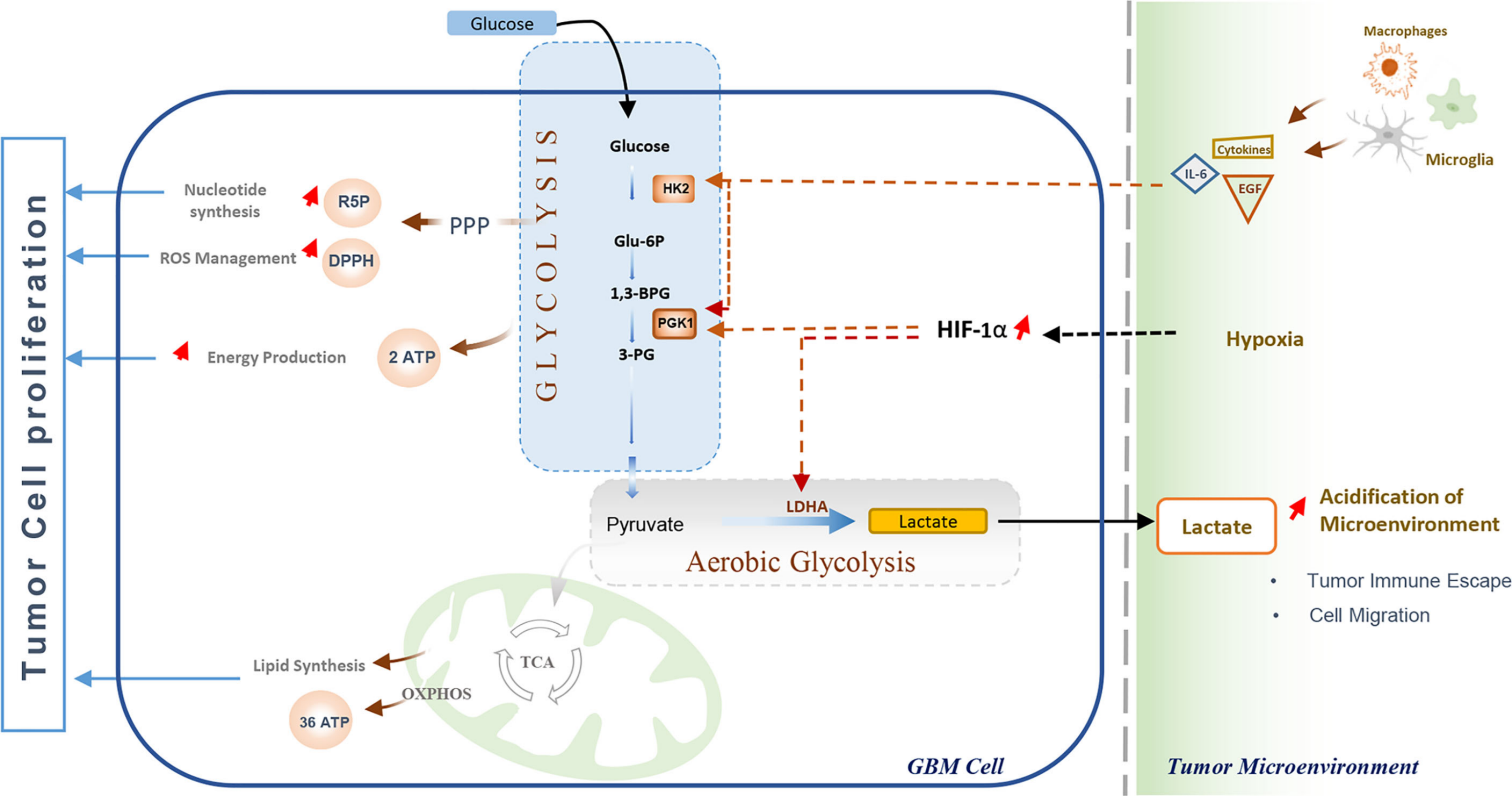
Glycolysis and its role in Glioblastoma cell proliferation and immune escape. The Warburg effect enables GBM cells to meet their energy demand through rapid ATP production, and promotes nucleotides synthesis and oxidative stress management, promoting cell proliferation. The elevated lactate production by tumor cells increases the acidification of the Tumor Microenvironment, which disturbs immune cells activation leading to impaired tumor immunosurveillance and cell migration. Immune cells (tumor-associated macrophages and Microglia) enhance glycolysis through Interleukin-6 (IL-6) and Epidermal Growth Factor (EGF), While hypoxic conditions in the Tumor microenvironment promote glycolysis and Warburg effect by activation and stabilization of hypoxia-inducible transcription factor (HIF-1α).

Rapid energy production has long been considered the Warburg effect’s main advantage in cancer cells. Currently, it is evidenced that cancer cells undergo aerobic glycolysis not only to meet their high energy demands but also as a critical adaptive process that enables cells to overcome the hypoxia conditions and effectively interact with other cells in the tumor microenvironment (95). Therefore, several metabolic pathways are ultimately activated to generate intermediate metabolites and macromolecules essential for cell proliferation or invasion (96, 97). In addition to ATP production, glycolysis fuels the pentose phosphate pathway by glucose-6-phosphate (G6P) and consequently accelerates the production of ribose-5-phosphate (R5P) and NADPH (96). These two molecules play an essential role in the adaptive metabolism of various cancers, including GBM (86). The ribose-5-phosphate (R5P) is an intermediate metabolite required for nucleotides synthesis, while NADPH acts as an antioxidant agent, combating high levels of intracellular ROS during cell proliferation. Additionally, NADPH is a crucial molecule for lipids biosynthesis, participating in 2-hydroxyglutarate (2‐HG) production in *IDH*-mutant glioblastoma (40). Further, it is thought to be involved inregulating apoptosis, invasion, and migration, thus promoting tumor cell proliferation and metastasis (98). Moreover, the increased lactate secretion resulting from aerobic glycolysis has stimulated angiogenesis and impaired tumor immunosurveillance in glioblastoma (97, 99) (**Figure 3**).

## Lipid metabolism in GBM

Recently, cancer lipid metabolism started to emerge as an opportunity to identify new therapeutic targets against various cancer types. Indeed, a growing body of studies started to explore how cancer cells support their tumorigenesis and cancer progression by reprogramming the metabolism of fatty acids, the most construction squares of several lipid species, counting phospholipids and triglycerides (100). Succeeding adipose tissues, the brain has the highest body lipid contents, which, beyond their vital role in building the brain structure, are also critical players as energy resources and as signaling molecules to maintain cell growth (101). However, the first study, performed by Brante et al. (1949), showed the presence of esterified cholesterols in brain tumors while absent in normal adult brain tissue (102). Since then, other studies have been conducted to analyze lipid composition in human intracranial tumors (103).

### Lipidomic Profile of GBM

Several *in vivo* and *in vitro* studies have been conducted to better understand the metabolic profile of lipids in GBM. Using GBM cell lines, it has been shown that the lipid profile of GBM cells changes in response to the nutrient composition. Moreover, the lipidomic analysis showed an accumulation, of five main classes of lipids, including Ceramides, Phospholipids, Diacyl-glycerols, Triacyl-glycerols, and Sphingomyelins (104). In contrast, in ectopic and orthotopic mouse xenograft models, lipidomic examination distinguished 500 lipid species, with the most prominent forms dropping transcendently into four primary categories: glycosphingolipids, glycerophosphoethanolamines, triacylglycerols, and glycerophosphoserines. Interestingly, contrasts were observed in lipid profiles when the same tumor was engendered within the flank fronting the brain, suggesting the fundamental significance of the encompassing physiological environment on GBM cancer growth (105). Moreover, lipid composition analysis of human glioma appeared a principal sum of phosphatidylinositol, sphingomyelin, and lysophosphoglycerides, in contracts to typical cortex tissue, as well as a noteworthy increase in oleic, linoleic, and arachidonic acid (106), cholesterol esters (103), and phospholipids (107).

### Lipid Droplet Metabolism

Lipid droplets (LDs) are intracellular organelles that regulate the storage and lipolysis of neutral lipids, such as triglycerides and sterol esters (108). Beyond their role of storage, deregulation of LDs metabolism has been associated with several types of tumor tissues from cancer patients (109). Recently, LDs have been raised as a potential biomarker of GBM. Most recently, using immunofluorescence, immunohistochemistry, and electronic microscopy, it has been shown that tumor tissues from GBM patients include massive amounts of LDs, which are not detectable in normal brain tissues and low-grade gliomas (110). Moreover, LDs prevalence was inversely correlated with GBM patient survival (110). In addition, nuclear magnetic resonance spectroscopy (NMR) revealed that triglycerides were present in biopsies from GBM brain patients but absent in healthy adult brain tissues (15). Moreover, a correlation has been established between lipid resonance spectra and the grade of malignancy (15). Alongside triglycerides, lipid droplets store cholesterol under their esterified form, cholesteryl ester, which is hydrolyzed by cholesteryl ester hydrolase, also known as hormone-sensitive lipase (111). Cholesterol Ester metabolism has also emerged as a promising pathway to target GBM treatment (9). Besides, *in vitro* studies have shown that densely plated astrocytes turn off the expression of genes associated with cholesterol synthesis and show a low cholesterol level, while glioma cells maintain a high cholesterol level (112).

### Fatty Acid Metabolism

In expanding *de novo* lipid synthesis, tumor cells can also use exogenous fatty acids to stimulate their growth. Recently, Acyl-CoA-binding protein, a regulator of LCFA intracellular metabolism in astrocytes very pronounced in GBM, reported controlling the accessibility of long-chain fatty acyl-CoAs to mitochondria, advancing fatty acid oxidation, tumor development, and poor survival through acyl-CoAs liaison, in several preclinical models (113, 114). Moreover, inhibiting fatty acid oxidation reduces proliferation in human glioma primary-cultured cells and prolongs survival in malignant glioma mouse models (115). On the other hand, as mentioned earlier, a mutation in *IDH*, the essential enzyme in the tricarboxylic acid cycle occurring in the mitochondria, is strongly associated with secondary GBM (116). Notably, a comprehensive metabolism investigation was performed on clinical *IDH1* mutant glioma specimens. The results showed a reduction in the pool of fatty acyl chains, displayed as a reduction in triglyceride and sphingolipid levels, without any change in membrane phosphatidyl lipids level (116).

**Figure 4:**
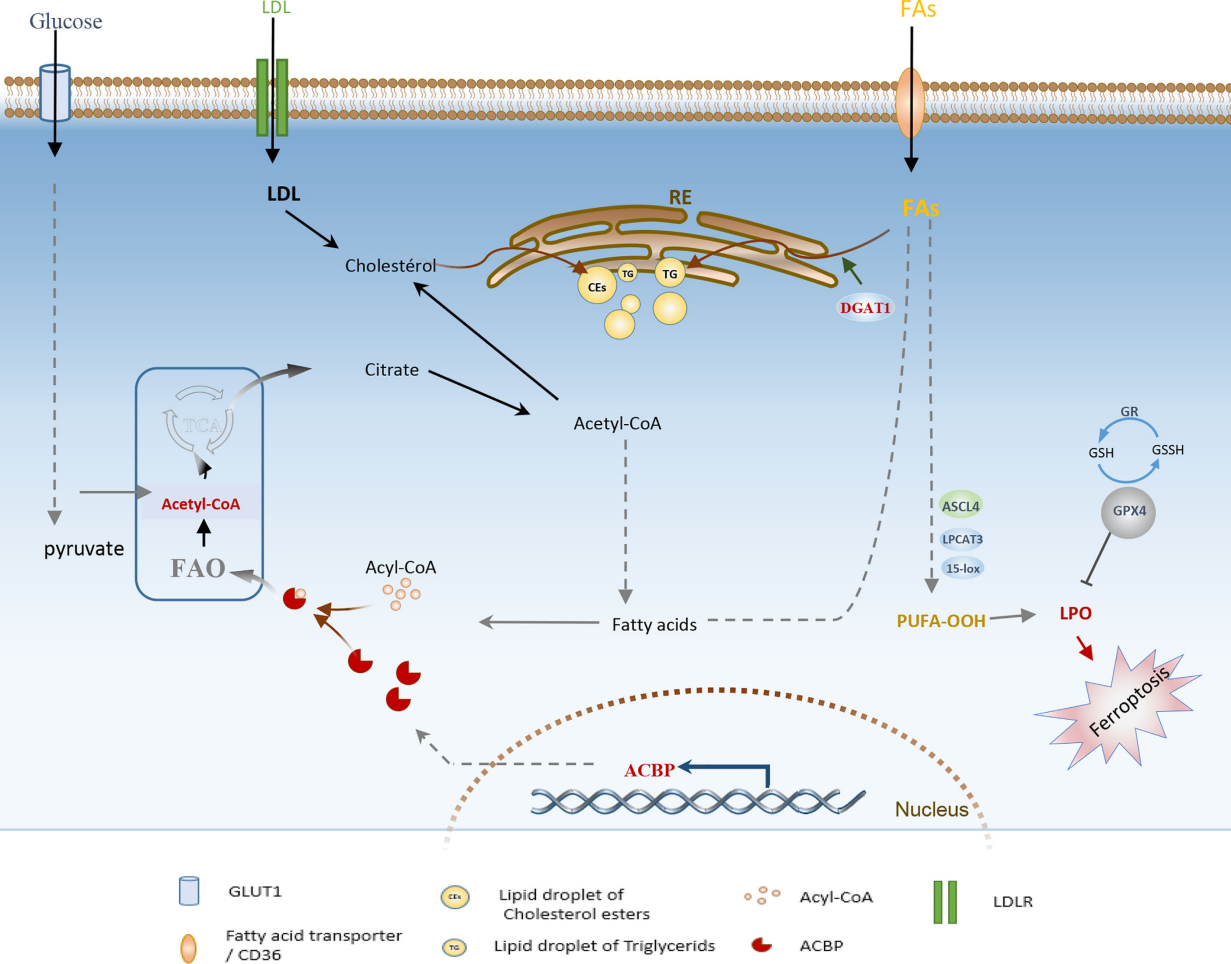
Schematic illustration of adaptive lipid metabolism in glioblastoma: role of the highly expressed ACBP, DGAT1, GPx4, and lipid droplets in lipid oxidation and ferroptosis induction. FAs, Fatty acids; FAO, Fatty acid oxidation; PUFA, Polyunsaturated fatty acids; DGAT1, diacylglycerol-acyltransferase 1; ACSL4, acyl-CoA synthetase long-chain family member 4; GPX4, glutathione peroxidase; GR, glutathione reductase; LD, lipid Droplet; LPO, lipid peroxides; ACBP, Acyl-CoA-binding protein; LDLR, low-density lipoprotein receptor; ER, Endoplasmic Reticulum.

The focus of recent research has been on the interplay between lipid metabolism and cell death mechanisms. However, an emerging research area is understanding specific lipid-associated metabolic dependencies of malignant cells and harnessing their potential to disrupt the lipid homeostasis, which triggers lipotoxicity and induces cell death. For example, Ferroptosis, a newly described form of programmed cell death due to the increased iron-associated lipid peroxidation and ROS production, has been recently investigated as a new direction in gliomas treatment (117). Indeed, several ferroptosis-associated genes were recently established, and targeting them using diverse approaches has shown significant outcomes in low-grade glioma and GBM. For instance, inhibiting diacylglycerol-acyltransferase 1 (DGAT1), which is known to catalyze the storage of excess FFAs into TGs and lipid droplets (**Figure 4**), has been shown to suppress GBM growth *in vitro* and in *vivo* by triggering oxidative stress and ferroptosis (118, 119). Similarly, depletion of glutathione peroxidase 4 (GPX4), an enzyme with a pivotal role in preventing phospholipid oxidation, was powerfully efficient in inducing ferroptosis in GBM (120, 121). Moreover, past studies have demonstrated that overexpression of acyl-CoA synthetase long-chain family member 4 (ACSL4) results from ferroptosis induction, yielding a remarkable effect on inhibiting glioma cell proliferation.

Even though the evidence demonstrates the critical role of lipid metabolism on tumorigenic properties of GBM, the lipid metabolic pathways are not yet completely understood, and results still derive from *in vitro* studies, mouse genetic models, and xenograft models. Understanding how GBM cells adapt their underlying molecular mechanisms to reprogram lipid metabolism depending on their environment will be the key to further translating primary findings to clinical use.

## Targeting gliomas metabolism - current therapeutic advances and ongoing clinical trials

Although the concept of targeting tumor metabolism is not entirely new, a better understanding of the metabolic landscape of malignant gliomas has opened the way for a growing number of investigations into the metabolic vulnerabilities of these tumors. Thus, considerable and continuous efforts are being directed toward exploiting specific dependencies to identify novel molecular targets and design new effective therapeutic strategies that help improve the patient’s clinical care with GBM (8, 35, 122). Indeed, the paradigm of focusing on cancer metabolism was freshly required beyond the dramatic flexibleness of tumor metabolic cascades reported in cancer cells challenged with multiplex inhibitors, including glycolysis and glutaminase inhibitors (123, 124).

However, recent research in gliomas and GBM clinical trials have focused mainly on the energetic aspect of cancer metabolism as a promising approach providing several potent targetable enzymes, aiming to overcome GBM heterogeneity and/or improve the outcomes of standard therapies. Currently, many drugs targeting glycolysis, OXPHOS, glutaminolysis, lipid, and nucleotide synthesis are being studied in clinical trials to treat gliomas (**Table 1**).

**Table 1:** Current Advances and main clinical trials in metabolism targeting for gliomas therapy.

Drugs or Therapeutics	Trial Phase	Target	Mechanisms	Gliomas type	References or Clinical Trial Identifier*
**IDH 305**	Phase II	Mutant IDH1	inhibitor of the mutant IDH 1 enzyme	Low Grade Glioma	NCT02987010 NCT02977689
**IDH 305**	Phase I	Mutant IDH1	mutant IDH small molecule inhibitor	Gliomas withIDH1R132 Mutations	NCT02381886
**AG-221**	Phase I/II	Mutant IDH2	inhibitor of the mutant IDH2 enzyme	Glioma, mutant IDH2	NCT02273739
**AG-120**	Phase II	Mutant IDH1	–	Glioma with an IDH1 mutation	NCT02073994
**AG-881**	Phase III	Mutant IDH1 or IDH2	Inhibitor of Mutant IDH1 and 2	Grade 2 Glioma	NCT04164901
**AG-120 and AG-881**	Phase I	Mutant IDH 1	Suppression of 2-HG	LGG	NCT03343197
**FT-2102**	Phase I/II	Mutant IDH 1	IDH1m Inhibitor	LGG, GBM	NCT03684811
**BAY1436032**	Phase I	Mutant IDH 1	IDH-R132X-inhibitor	Anaplastic glioma, GBM	NCT02746081
**Metformin**	Early Phase I	AMPK/Mitochondrial complex I	Metformin blocks oxidative phosphorylation in mitochondria	GBM	NCT03151772
	Phase II			GBM	NCT02780024
	Phase I/II			IDH1/2 Gliomas	NCT02496741
	Phase I			GBM	NCT01430351
**CB-839**	Phase I b	Glutaminase (GLS)	Chemical inhibitor	Diffuse Astrocytoma, IDH-Mutant	NCT03528642
**TVB-2640**	Phase II	Fatty-acid synthase (FASN)	VB-2640 inhibits the β-ketoacyl reductase (KR) enzymatic activity of the FASN	High Grade Astrocytoma	NCT03032484
**PEG-BCT-100**	Phase I/II	Arginine	Depletion of circulating arginine	High Grade Gliomas	NCT03455140
**Methotrexate**	Phase II	Dihydrofolate reductase (DHFR)	Inhibition of folate metabolism and nucleotide synthesis	Glioblastoma multiforme	NCT00082797
**Dichloroacetate (DCA)**	Phase II	Mitochondrial PDHK	DCA switches metabolism from the cytoplasmic glycolysis to the mitochondrial glucose oxidation	Glioblastoma multiforme	NCT00540176
**Ubidecarenone** **BPM31510**	Phase I	Redox toxicity elevation in mitochondrial O2− species	BPM31510 works by correcting cancer cell metabolism,	Glioblastoma multiforme	NCI-2016-01973
**ONC201**	Phase II	Caseinolytic protease P (ClpP)	ONC201 acts as a ClpP which regulates oxidative phosphorylation	Adults With EGFR-low Glioblastoma	NCT04629209
	Phase I		Targets OXPHOS and suppresses mitochondrial respiration	Pediatric H3 K27M Gliomas	NCT03416530
	Phase II		-	Recurrent H3 K27M-mutant Glioma	NCT03295396
**Perifosine KRX-0401**	Phase II	AKT/PI3K	inhibitor of Akt and PI3K, modulates phospholipid metabolism	Malignant Gliomas	NCT00590954
**Atorvastatin**	Phase II	HMG-CoA reductase	Inhibition of cholesterol biosynthesis	Glioblastoma	NCT02029573
**2-DG**	Phase I/II	Glycolytic enzyme	Glucose analog	Malignant Gliomas	(125)

Many current metabolism-based therapies against gliomas have attempted to harness the fact that IDH mutations are among the most common alterations that drive metabolism remodeling in low-grade gliomas and secondary GBM (44). Therefore, numerous researchers considered isocitrate dehydrogenase as an actionable target and have been performed over the past decade to take advantage of the inhibition of mutant IDH enzymes through pharmacological inhibitors or small molecules to disrupt tumor metabolism and alter thereby tumor growth. As a result, multiple IDH-selective inhibitors have been advanced to focalize tumors harboring IDH mutations. For instance, AG-881, a chemical inhibitor of IDH1, is the foremost enhanced IDH inhibitor for grade II gliomas that have launched a phase III clinical trial with a significant outcome in this grade of gliomas. In line with this, IDH-mutant glioma cells have been indirectly inhibited by (AGI-5198), an IDH1 inhibitor known to act specifically against R132H alterations (126, 127).

Currently, other clinical trials have discovered novel inhibitors targeting both IDH1 and IDH2. Thus, allowing the control of other malignancies harboring IDH mutations, such as gliomas invasiveness, has been shown, mechanistically, to decrease 2-HG levels (128). In this regard, IDH305 (Novartis) targets IDH1 mutations, and ongoing trials are presently being conducted in gliomas and other malignancies with IDH1 R132 mutations (NCT02381886). In addition, FT2102 (Forma Therapeutics) and BAY1436032 (Bayer) also target IDH1 R132 tumors (R132X for BAY1436032), and occurring trials are selecting patients with various tumor forms, including GBM (NCT03684811 and NCT02746081).

Other inhibitors are being developed to manage glioma tumors in the same context. Among these, AG221 (Enasidenib, CC90007; Agios) reported targeting IDH2 mutations and is currently beneath improvement to treat gliomas, in addition to AG881 (a pan-IDH1/-2 (Agios) inhibitor which is being developed for the administration of gliomas cancers. This molecule restrains the cytoplasmic IDH1 and IDH2 within the mitochondria; AG-881 can also penetrate the blood-brain barrier.

Undoubtedly, glutamine metabolism can indirectly influence IDH1/2 function since glutamine-derived glutamate is a precursor of ketoglutarate, the amounts of which determine the rate of IDH1/2 activity (123). In fact, beneath certain metabolic circumstances, such as hypoxia, cancer cells preferentially employ glutamine-derived chemicals in lipogenesis over the preferred IDH1 pathway-producing molecule (58, 129). Patients with IDH-mutated GBM may benefit from glutamine metabolism targeting since it will efficiently disrupt *de novo* lipids synthesis, crucial to sustaining cancer cell proliferation and tumor growth.

Besides clinical trials targeting directly mutant IDH enzymes, a wide range of emerging assays has been alternatively attempting to harness other metabolic aspects by acting on different effectors such as mTOR and EGFR, which are extensively targeted using various classes of molecules, including monoclonal antibodies (mAbs) and tyrosine kinase inhibitors (TKI) (130). Similarly, several metabolic enzymes have emerged and clinically tested, notably those involved in glucose oxidation, oxidative phosphorylation, fatty acids, and nucleotide synthesis. For instance, a phase II clinical trial has been led to evaluate the safety and efficiency of Dichloroacetate (DCA), which is an inhibitor of the mitochondrial pyruvate dehydrogenase kinase that is believed to shift metabolism from the cytoplasmic glycolysis to mitochondrial glucose oxidation, thereby altering the GBM metabolism and inhibiting the resistance to apoptosis (131).

Up to this point, the metabolic-driven treatment strategies had been challenging to implement clinically and had no longer proven effective thus far. Consequently, there may be a growing need to test innovative metabolism-driven approaches that target diverse elements of glioma metabolism, including glutamate-glutamine metabolism, for example, the utilization of a systemic glutaminases inhibitors therapy.

Of note, glutamine metabolism targets are also being developed as therapeutic approaches. For instance, targeting glutaminase (GLS), which catalyzes the primary stage of glutamine metabolism, leads to the development of a new drug, the CB-839 hydrochloride (CB-839), which is currently in phase 1b trial. This trial focused on combining CB-839 with radiotherapy and temozolomide to evaluate the efficacy and safety of treating IDH-mutated diffuse astrocytoma. The election of the most relevant players in glutamine metabolism will assist in the layout of novel treatments that might lethally disturb GBM cells and impair disease development.

## Recent *in vitro* and *in vivo* studies highlight new potential metabolic targets for glioblastoma therapy

In addition to all anterior researched targets clinically tested, recent evidence from preclinical models and *in vitro* studies reveals many new potential metabolic targets for glioblastoma, showing encouraging results with a remarkable inhibitory effect on tumor growth and malignancy (8, 9, 88, 119, 132). Notably, among the proposed actionable targets, several glycolytic enzymes such as hexokinase2 (HK2), phosphofructokinase-1 (PFK1), and phosphoinositide-dependent kinase-1 (PDK1) were considered for therapeutic use and experimentally evaluated (**Table 2**). Further, previous *in vitro* and *in vivo* studies have noted that targeting OXPHOS proteins, lipoxygenases, and many other metabolic effectors involved in cholesterol and amino acid metabolism, fatty acids, and nucleotide synthesis, have been able to disrupt the altered metabolism of GBM, yielding significant results that should be harnessed for better therapeutic solutions (8, 152).

**Table 2:** Potential targets and promising agents against glioblastoma metabolism.

Potential Targets	Therapeutic approaches	Study type	Mechanism of action	References
**OXPHOS Proteins** **(complex V)**	Gboxin	*In vitro* and *in vivo* study	inhibits the activity of F0F1 ATP synthase and thus disrupt cell metabolism	(133)
**(MCT1)**	AR-C117977 And others	*In vitro* study	Intracellular acidification and induction of cell death	(134)
**LPCAT1**	LPCAT1-shRNA	*In vivo* study	Suppression of EGFR signaling and alteration of membrane lipid remodeling	(135)
**PHGDH**	inhibition of PHGDH with CBR-5884	*In vitro* study	Alteration of Serine metabolism	(136)
**SOAT1**	shRNA mediated *LPCAT1* knockdown	*In vivo* study	Block SREBP-1-mediated lipogenesis	(110)
**(ACAT-1)**	Inhibition by Avasimibe	*In vitro* and *in vivo* study	Decrease cholesteryl ester storage in lipid droplets and increase intracellular free cholesterol balances	(137)
**Glycolytic enzymes**	3-bromopyruvate (3BP) (An antiglycolytic agent)	*In vivo* study	3BP induces alterations in proteins involved in aerobic glycolysis and carbohydrate metabolism.	(138)
**HK2**	ketoconazole and posaconazole (HK2 inhibitors)	*In vitro* and *in vivo* study	reduce tumor growth likely *via* blocking HK2 and affecting tumor metabolism	(139)
**HK2 and** **miR-218**	shRNA mediated HK2 silencing/ miR-218 overexpressing	*In vitro* and *in vivo* study	miR-218 overexpression downregulates HK2, inducing thereby glycolytic metabolism alteration and cell death.	(140)
**LXR**	Synthetic LXR agonist (LXR-623)	*In vitro* and *in vivo* study	LXR-623 kills GBM cells by reducing cellular cholesterol through activation of LXRβ.	(141)
**ACBP**	shRNA mediated *ACBP* knockdown	*In vivo* study	Reduction of ACBP expression decreases Fatty acid oxidation and hinder GBM cell proliferation.	(114)
**SREBP-1, FAS and FDFT1**	Phytol (PHY) and retinol (RET)	*In vitro* study	PHY and RET modulates cholesterol and fatty acid biosynthetic pathways.	(142)
**Cholesterol metabolism (SREBP-1, LXR-α, HMG-CR and LDLR…)**	27-Hydroxycholesterol (27-OHC)	*In vitro* study	27-OHC inhibits cholesterol synthesis and promote its transport.	(143)
**miR-448–HIF-1α axis**	HIF-1α signaling	*In vitro* study	miR-448 negatively regulates HIF-1 α signaling	(144)
**PFK1 and PDK1**	Gene Knockdown	*In vivo* study	Inhibitions of glycolysis and alteration of cellular metabolism	(145)
				
**Glucose analogs**	2DG, a glucose analog and glycolytic inhibitor	*In vitro* study	In combination with metformin Inhibits Proliferation and Cellular Energy Metabolism and induces ER stress in	(146, 147)
**Energy Depletion**	Dual MR *via* ONC201/TIC10 and 2-Deoxyglucose	*In vitro* study	Targeting of OXPHOS *via* ONC201/TIC10 suppresses mitochondrial respiration and 2DG inhibits glycolysis, leading to energy depletion and enhanced anti-cancer activity	(132)
**HSPD1**	Synthetic small molecule (KHS101)	*In vitro* and *in vivo* study	Alteration of Mitochondrial bioenergetic capacity and glycolytic activity	(148)
**NAD+**	NAMPT inhibitors (GMX1778) NAMPT knockdown	*In vitro* and *in vivo* study *In vitro* study	NAD+ depletion alter tumor microenvironment Reduction of cell proliferation, migration, and invasion and induction of apoptosis	(149, 150)
**Lipoxygenase** **(15-LOX)**	15-Lipoxygenase Inhibitors (Luteolin and NDGA)	*In vitro* study	15-LOX inhibition reduced migration and raised cell cycle arrest in the G2/M phase	(151)

While mutated genes, especially those encoding metabolic enzymes and other vital metabolites such as the IDH1/2, were widely investigated in several malignancies, recent evidence highlights the relevance of unmutated forms of these enzymes in metabolic alterations as a hallmark of cancer additionally. Calvert et al., for instance, demonstrated *via in silico* and wet-bench investigations that non-mutated isocitrate dehydrogenase 1 (IDH1) is often overexpressed in primary GBM and that genetic and pharmacological inactivation leads to reduction of GBM tumor growth (153). Therefore, rather than focusing exclusively on mutated genes, new treatment approaches and future research should consider investigating and addressing the unmutated enzymes’ implications and potential.

Given that various cancers share a broad spectrum of genomic alterations, most metabolic abnormalities and actionable targets recently evidenced in glioblastoma are likely to be non-tumor specific (154). Moreover, the heterogeneous nature of GBM is another challenge in the way of establishing efficient metabolic-based therapeutic approaches. Thus, focusing on specific genetic mutations and their downstream metabolic pathways may offer great opportunities for more targeted therapy. For instance, in low-grade gliomas, the IDH1/2 is a significant target. A dozen clinical trials have been conducted to explore its potential in IDH-mutant gliomas (**Table 1**). In addition, *EGFR* amplification and *EGFR*vIII, a tumor-specific mutations frequently reported in glioblastoma, have been demonstrated to be involved in metabolism remodeling and recently regarded as promising clues for metabolism-based therapy in GBM (47). On the other hand, the emerging link between cancer genetic alterations and metabolic dependencies raises the critical need for coupling new metabolic therapeutics with specific biomarkers to deliver more targeted personalized treatments.

Overall, dysregulated metabolic pathways in glioblastoma offer considerable opportunities to improve treatment outcomes, whereas developing effective approaches is still challenging. However, small-molecule inhibitors and monoclonal antibodies remain the main therapeutic strategies being used so far, and many of them are undergoing clinical trials intending to inhibit key metabolic targets in gliomas. Genome editing and RNA therapy have recently emerged as powerful tools to screen targetable genes and understand the molecular mechanisms underlying the pathological phenotype and can be exploited for new therapeutic advances (155). Indeed, research in metabolism-targeting has benefited from the emerging technology of CRISPR/Cas9 and RNA therapeutics (ASO, siRNA, and shRNA). Meanwhile, recent studies on GBM cell lines and animal models have applied these targeted tools to establish the proof-of-concept and assess the efficiency of several metabolic candidates. Nevertheless, these versatile strategies remain lacking from ongoing clinical studies.

## Conclusion

In conclusion, emerging evidence linking genetic and epigenetic alterations in cancer to altered metabolism has significantly enhanced our understanding of metabolic rewiring, a hallmark of gliomas tumors. Indeed, GBM cells shift their energetic metabolism towards a variety of metabolic states characterized by rapid and enhanced ATP generation as well as increased anabolic metabolism (8, 92). These changes help cells sustain the high proliferation rate and adapt to continuous fluctuations in the tumor microenvironment, particularly changes in nutrients and oxygen availability (95–97). Besides, numerous studies suggested a key role of these metabolic adaptations in immune cell dysfunction and cancer immune escape *via* acidification of the TME, secretion of immunosuppressive cytokines, and induction of epigenetic modifications in immune cells (156–158). Alongside this, similar metabolic reprogramming occurs on the surrounding immune cells, affecting their activation and function, altering the anti-cancer immunity, and thereby enabling tumor cells to evade the immunosurveillance system (159). Additionally, the altered metabolic landscape is tightly linked to the ability of tumor cells to maintain the cellular redox balance and escape apoptosis under tumor conditions (43, 58).

Importantly, GBM cells are metabolically heterogeneous within the tumor. They display distinct metabolic profiles and intrinsic plasticity to hypoxic and nutritionally deficient conditions through compensatory enhancement of alternative metabolic pathways (91, 160). In this sense, therapeutic approaches based on co-targeting several metabolic dependencies may represent a powerful strategy to tackle this dynamic metabolic nature of GBM cells.

From a therapeutic perspective, dysregulated metabolic pathways in GBM offer considerable opportunities to improve treatment outcomes, while the development of effective approaches is still lacking. However, several challenges, such as the rapid and continuous alterations at the genetic and epigenetic level leading to increased crosstalk between pathways, have made such therapies non-ubiquitous. This ending raises the need for exploring and exploiting new horizons in GBM metabolism. In this review, we focused on the most common and well-characterized genetic patterns affecting glioma’s cell metabolism. However, more research is being conducted to explore the relevance and the potential of other effectors, notably non-coding RNAs and epigenetic alterations, as new and promising aspects that may offer exploitable vulnerabilities to disrupt GBM metabolism with translational implications (161–164).

## Author Contributions

Conceptualization: RE and AE; Methodology: RE, AE, NB, BT, ZW, and AZ; Writing: All authors; Supervision: RE. Funding acquisition: RE. All authors contributed to the article and approved the submitted version.

## Funding

This work received UM6P’s internal welcoming funds and the FFI from OCP.

## Conflict of Interest

The authors declare that the research was conducted in the absence of any commercial or financial relationships that could be construed as a potential conflict of interest.

## Publisher’s Note

All claims expressed in this article are solely those of the authors and do not necessarily represent those of their affiliated organizations, or those of the publisher, the editors and the reviewers. Any product that may be evaluated in this article, or claim that may be made by its manufacturer, is not guaranteed or endorsed by the publisher.
